# Biological activities and phenolic compounds of olive oil mill wastewater from Abani, endemic Algerian variety

**DOI:** 10.1038/s41598-022-10052-y

**Published:** 2022-04-11

**Authors:** Zakia Gueboudji, Dalila Addad, Kenza Kadi, Kamel Nagaz, Mansour Secrafi, Leila Ben Yahya, Belgacem Lachehib, Assia Abdelmalek

**Affiliations:** 1 Biotechnology, Water, Environment and Health Laboratory, Abbes Laghrour University of Khenchela, Khenchela, Algeria; 2 Faculty of Nature and Life Sciences, Abbes Laghrour University of Khenchela, Khenchela, Algeria; 3 Drylands and Oases Cropping Laboratory, IRA Medenine, Medenine, Tunisia

**Keywords:** Biochemistry, Biological techniques, Biotechnology

## Abstract

The current study aimed to determination of cytotoxicity, antioxidant, anti-inflammatory, anti-hemolytic, and anticoagulant activities of phenolic compounds extracted from olive oil mill wastewater (OMW) issue from the cold extraction of olive oil from Khenchela eastern in Algeria. The LC–MS (liquid chromatography–mass spectrometry) results were revealed the presence of 20 phenolic compounds in the extract of OMW and mostly consisted of Kaempferol, 4,5-di-O-caffeoyquinic acid, quinic acid, and caffeic acid. The extracts possessed effective reducing power (FRAP) and high radical scavenging activity against DPPH (2,2-diphenyl-1-picrylhydrazyl), ABTS + (2,2′-azino-bis (3-ethylbenzothiazoline-6-sulfonic acid) free radicals, and it inhibited cytochrome c reduction in a dose-dependent manner. They exert a protective effect on red blood cells, and they were found to exhibit the highest inhibitory effect anti-inflammatory activity using inhibition of protein denaturation (IPD) and membrane stabilizing potential (MSP) tests (80.46 ± 3.81 µg/mL and 87.43 ± 0.66 µg/mL) more than the standard used. The extract also showed the greatest anticoagulant activity in both the endogenous and exogenous routes (44.77 ± 0.25 s and 15.84 ± 0.12 s, respectively). Based on these findings, it is reasonable to infer that OMW is a good source of natural phenolic compounds with potential antioxidant, anti-inflammatory, and anticoagulant properties.

## Introduction

The olive mills activity creates huge quantities of waste in a short period, generally 4 months from October to January. Based on the extraction methods used, an estimated average volume of olive mill wastewater (OMW) ranging from 0.3 to 1.2 m^3^/tons of processing olives. Also, an average quantity of solid residue ranging from 500 to 735 kg/tons of processing olives has been observed^1^. The OMW is a turbid, watery, black, and foul-smelling liquid that contains easily fermentable emulsified grease^2,3^. It has a toxic effect on soil, microorganisms, plants, and marine organisms^4,5^. The OMW comprised about 98% of the total phenolic content of olive fruits ranging from 0.5 to 24 g/L^6^, including hydroxytyrosol, tyrosol, and flavonoids^7^. Pollution is mainly due to the high concentrations of phenolic compounds^8^. Because of their acidity, high levels of biological oxygen demand, and chemical oxygen need, OMWs are highly contaminating and phytotoxic. On the other hand, OMWs, are a valuable source of molecules such as plant nutrients, anthocyanins, flavonoids, polysaccharides, and several phenolic compounds with industrial applications such as fertilizers, antioxidants, antifungal and antibacterial drugs, cytoprotective agents, gelling and stabilizing agents in food preservation. As a result, significant efforts have been made to shift from OMW detoxification to its commercialization by maximizing the recovery of high added-value bioactive chemicals^9^. Phenolic compounds have many biological properties, particularly antioxidant, anti-inflammatory, and anticoagulant effects, which are used in the pharmaceutical industries^10^.

Cell damage caused by free radicals has been linked to several diseases including cancer, arthritis, and diabetes. Some free radicals and reactive nitrogen species can activate and upregulate cell death pathways such as apoptosis. Antioxidants have been linked to a range of therapeutic properties. Several researchers have hypothesized that antioxidants might reduce the harmful free radical effects improving consequently the therapeutic effectiveness^11^. However, oxidative stress is unsteady in the balance between the defense system of antioxidants and the production of reactive oxygen species (ERO)^12^. This causes biochemical damage in the organism's cells due to molecular repercussions, such as changes in proteins, the appearance of DNA breaks, or damage to the cell membrane integrity owing to the induction of lipid peroxidation. Oxidative stress has been described as a crucial etiological factor involved in various chronic human diseases such as cancer, cardiovascular and neurodegenerative diseases, inflammation, diabetes mellitus, and aging^13^.

Inflammation and coagulation are two major host defense mechanisms that work in tandem^14^. They are implicated in many cardiovascular diseases such as thrombosis and atherosclerosis^15^. Thromboembolic diseases continue to be the leading cause of death throughout the world^16^. As is well known, thrombosis is closely related to activating platelet adhesion, aggregation, secretion functions, and activation of intrinsic and extrinsic coagulation systems, which cause blood coagulation and fibrin formation^17^. Therefore, anticoagulants play a pivotal role in the prevention and treatment of thrombotic disorders^18^. Diseases arising from blood clotting, including pulmonary emboli, deep vein thrombosis, and cardiovascular diseases are the main causes of death and disability worldwide^19^.

Thrombosis is a process in which a blood clot develops within a blood artery and can obstruct blood flow in the afflicted area, eventually creating disorders such as pulmonary emboli, deep vein thrombosis, strokes, and heart attacks. On the other hand, Thrombolytic agents are those that inhibit the development of a blood clot in blood arteries, such as tissue plasminogen activator (tPA), streptokinase (SK) urokinase, and others. Natural thrombolytic medicines derived from traditionally significant medicinal plants may be a viable source in this case, as they have few negative effects than manufactured pharmaceuticals. Nowadays, more than 60% of cytotoxic agents are derived from natural sources such as plants, marine creatures, and microbes, either directly or by chemical modification based on natural lead compounds. Furthermore, natural compounds have a wide range of applications in cancer treatment^20^. Hence, the main objective of the current report is to evaluate the cytotoxicity, antioxidant, anti-inflammatory, and anticoagulant activities of phenolic compounds of OMW obtained from cold extraction of olive oil from Khenchela eastern Algeria.

## Results

### Physicochemical properties

The physicochemical characteristics of OMW that were studied are summarized in Table 1. The OMW is an acidic liquid effluent (pH = 4.9 ± 0.01) loaded with organic and mineral materials as indicated by a high electrical conductivity value (EC = 12.89 ± 0.09 mS/cm). The total suspended solids, biological (BOD_5_) and chemical (COD) oxygen demands were respectively 0.9%, 68 and 170 g/L. The dry (DM), organic (OM) and mineral (MM) matters were 110.8 and 53.7 g/L respectively.

**Table 1:** Physicochemical properties of OMW studied.

pH	EC (mS/cm)	TSS%	DM (g/L)	OM (g/L)	BOD_5_ (g/L)	COD (g/L)
4.9 ± 0.01	12.89 ± 0.09	0.9 ± 0.03	110.8 ± 3.17	53.7 ± 1.16	68 ± 2.28	170 ± 8.5

### Total phenolic, flavonoids and tannins content

The results of total phenolic, flavonoids and condensed tannin contents were summarized in Table 2. The Student's t-test revealed a significant difference between the means of the phytochemical contents. According to the findings, OMW is distinguished by a high polyphenol (10.82 ± 0.11 mg GAE/mL), flavonoid (3.11 ± 0.16 mg RE/mL), and condensed tannin (2.43 ± 0.15 mg TAE/mL) contents.

**Table 2:** Total polyphenols, flavonoids and condensed tannins of phenolic extract of OMW.

Secondary metabolites	Total polyphenols (mg GAE/mL)	Total flavonoids (mg RE/mL)	Condensed tannins (mg TAE/mL)
Concentration	10.82 ± 0.11	3.11 ± 0.16	2.43 ± 0.15

### LC–MS separation and identification of phenolic compounds

The quantitative analysis results of major phenolic compounds identified in the of OMW extract were summarized in Table 3. Liquid chromatography–mass spectrometry (LC–MS) was used to screen thirty-one (31) compounds. In the extract, only twenty (20) compounds were identified including 8 phenolic acids and 12 flavonoid compounds. The 4,5-di-*O*-caffeoyquinic acid (676.57 µg/mL), protocatechuic acid (195.35 µg/mL), quinic acid (194.53 µg/mL), gallic acid (65.92 µg/mL), salviolinic acid (33.82 µg/mL), caffeic acid (29.26 µg/mL), and p-coumaric acid (21.93 µg/mL) were classified as the most abundant phenolic acids. Among the individual flavonoid compounds in OMW extract, five were detected as predominant: kaempferol (906.83 µg/mL), apigenin (96.2 µg/mL), cirsiliol (51.5 µg/mL), naringin (16.72 µg/mL), and luteolin-7-*O*-glucoside (15.44 µg/mL).

**Table 3:** LC–MS analysis of phenolic extract of OMW.

No	Phenolic compounds	Chemical class	Rt (min)	MW	Ionization forms	Concentration (µg/L)
1	Quinic acid	Phenolic acid	2.03	191	[M–H]^−^	194.53 ± 29.092
2	Gallic acid	Phenolic acid	4.10	169	[M–H]^−^	65.925 ± 91.78
3	Protocatechuic acid	Phenolic acid	7.40	153	[M–H]^−^	195.35 ± 58.578
4	Caffeic acid	Phenolic acid	15.98	179	[M–H]^−^	29.26 ± 9.519
5	*p*-coumaric acid	Phenolic acid	22.11	163	[M–H]^−^	21.93 ± 5.604
6	Rutin	Flavonol	24.93	609	[M–H]^−^, [2M–H]^−^	2.106 ± 0.445
7	Trans-frolic acid	Phenolic acid	24.28	193	[M–H]	2.708 ± 4.69
8	Hyperoside (quercetin-3-*O*-galactoside	Flavonol	25.07	463	[M–H]^−^, [2M–H]^−^	4.801 ± 0.655
9	Luteolin-7-*O*-glucoside	Flavone	25.67	447	[M–H]^−^	15.445 ± 1.365
10	Naringin	Flavanone	27.04	579	[M–H]^−^, [2M–H]^−^	16.721 ± 0.532
11	4,5-Di-*O*-caffeoyquinic acid	Phenolic acid	27.64	515	[M–H]^−^	676.57 ± 83.712
12	Quercetrin (quercetin-3-*O*-rhamonosid	Flavonol	27.92	447	[M–H]^−^, [2M–H]^−^	12.310 ± 1.248
13	Apegenin-7-*O*-glucoside	Flavonol	27.90	431	[M–H]^−^, [2M–H]^−^	0.732 ± 1.269
14	Salviolinic acid	phenolic acid	28.918	717	[M–H]^−^	33.82 ± 2.943
15	Kaempferol	Flavonol	32.87	285	[M–H]^−^, [2M–H]^−^	906.831 ± 306.164
16	Quercetin	Flavonol	32.87	301	[M–H]^−^, [2M–H]^−^	2.457 ± 0.291
17	Naringenin	Flavanone	34.78	271	[M–H]^−^, [2M–H]^−^	1.367 ± 2.368
18	Apigenin	Flavone	35.42	269	[M–H]^−^, [2M–H]^−^	96.2 ± 10.05
19	Cirsiliol	Flavone	36.46	329	[M–H]^−^	51.258 ± 2.664
20	Cirsilineol	Flavone	39.01	343	[M–H]^−^	2.924 ± 5.065

### DPPH^+^, ABTS^+^ and FRAP assays

The antioxidant potential of the studied extract are summarized Table 4. The DPPH., ABTS.+, and FRAP free radical scavenging activity is frequently utilized to evaluate the antiradical/antioxidant capability of OMW phenolic compounds and compared the results using a variety of reference standards in order to obtain more useful and perhaps required findings. The findings of DPPH scavenging revealed that the phenolic extract of OMW had the highest antioxidant activity (IC_50_ = 9.62 ± 0.28 g/mL), which was comparable to rutin (IC_50_ = 10.5 ± 0.36 μg/mL) and greater than BHT and ascorbic acid (20.03 and 20.84 μg/mL). Additionally, the OMW possessed high scavenging activities against the ABTS free radical which evidenced by low IC_50_ value (7.10 μg/mL). Moreover, the extract exhibited the highest ferric reducing power (IC_50_ = 3.59 ± 0.24 µg/mL) than rutin (IC_50_ = 4.72 µg/mL), ascorbic acid (11.08 µg/mL) and BHT (IC_50_ = 18.81 µg/mL).

**Table 4:** Antioxidants activity of phenolic extract of OMW by DPPH, ABTS, and FRAP.

	DPPH (IC_50_ μg/mL)	ABTS (IC_50_ μg/mL)	FRAP (IC_50_ μg/mL)
Extract	9.62 ± 0.28^d^	7.10 ± 0.11^b^	3.59 ± 0.24^d^
BHT	20.03 ± 0.25^b^	4.27 ± 0.38^c^	18.81 ± 0.09^a^
Ascorbic acid	20.84 ± 0.65^a^	2.03 ± 0.14^d^	11.08 ± 0.18^b^
Rutin	10.5 ± 0.36^c^	8.48 ± 0.33^a^	4.72 ± 0.21^c^

### Cytotoxicity effect

#### Brine shrimp cytotoxicity test

The statistical analysis results revealed a very highly significant difference between the percentages of larval mortality concerning the phenolic extract and the K_2_Cr_2_O_7_ standard with *p* < 0.001. The results of larval toxicity by "brine shrimp" test of phenolic extracts of OMW studied are presented in Table 5.

**Table 5:** Brine shrimp lethality bioassay of phenolic extract of OMW and K_2_Cr_2_O_7_ used as standard.

Concentration (µg/mL)	% of Death of OMW	% of Death of K_2_Cr_2_O_7_
0	0	0
1	5.0 ± 0.1^e^	10 ± 0.6^f^
5	10 ± 0.1^d^	20 ± 0.5^e^
10	42 ± 0.31^c^	55 ± 0.3^d^
20	80 ± 0.2^b^	60 ± 0.2^c^
40	100 ± 0.3^a^	80 ± 0.5^b^
80	100 ± 0.1^a^	100 ± 0.1^a^
LC_50_	23.72 ± 0.1	33.74 ± 0.1

*LC*_*50*_ lethal concentration of 50% mortality.

The results reveal that the phenolic extracts of OMW have positive lethal effects on the larvae (nauplius) of brine shrimp after 24 h of exposure to the extracts; indicating that they are biologically active with mortality percentages gradually increasing with increasing frequency increase in the concentration of the extracts studied.

**Figure 1:**
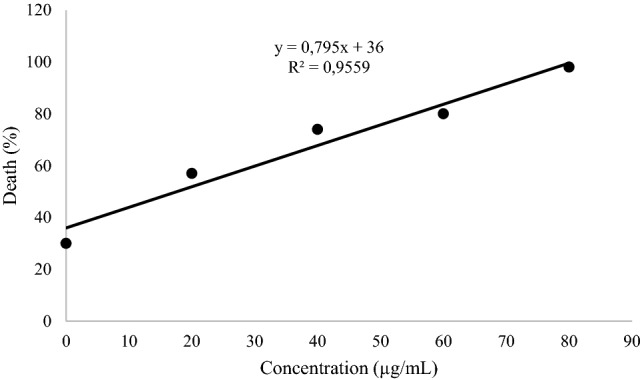
Lineal curve representing the percentage of mortality of *Artemia salina* according to the concentrations of OMW.

Figure 1 showed that the larvae were sensitive to OMW phenolic extracts according to a dose–response relationship. The DC_50_ values ​​were obtained from the regression equation of the linear curve of the percentage mortality of shrimp nauplii as a function of the concentrations phenolic extract of OMW tested, including the DC_50_ equal to (23.72 ± 0.1 μg/mL) that showed the lowest concentration compared to the standard used which gave a DC_50_ (33.74 ± 0.1 µg/mL).

#### Cytotoxicity test of the phenolic extract of OMW on human neutrophils

The viability of neutrophils is an essential test if it has been plan to use molecules in humans. To test the purified extracts, the purity of the freshly isolated neutrophils is checked with crystal violet (dye-based on gentian violet and acetic acid) and they are counted. Cells are suspended for the second time in phosphate buffer and stored at 4 °C before using them. Before testing the extracts of the polyphenols on the neutrophils, the cytotoxicity of the product is checked by incubating the neutrophils in the presence of high concentrations of polyphenols ranging from 0 to 300 μg/mL for 30 min, the dead cells allow the blue of trypan, which stains them blue, unlike living cells which remain transparent.

The results (Table 6) showed that the phenolic extract had no toxic effect on neutrophils, even at higher concentrations; the neutrophil viability rate exceeds 95%, so the rate of dead cells is not significant. The obtained solvent-free extract shows that no toxicity to cells; the trypan blue exclusion test showed a viability average greater than 96% even at high concentrations.

**Table 6:** Cytotoxicity test of phenolic extract of OMW on human neutrophils.

Phenolic extract (µg/mL)	% viability of PMNs
0	100
50	98
100	96
200	95
300	98

### Anti-inflammatory activity

#### Inhibition of protein denaturation (IPD)

Table 7 present the findings of the denaturing effect of proteins. The studied phenolic extract of OMW has an inhibitory efficiency of thermal denaturation (IC_50_ = 80.46 ± 3.81 µg/mL), superior to that of the reference anti-inflammatory drug diclofenac sodium (IC_50_ = 83.83 ± 0.21 µg/mL).

**Table 7:** Anti-inflammatory activity of phenolic extract of OMW.

	IPD IC_50_ µg/mL	MSP IC_50_ µg/mL
Extract	80.46 ± 3.81^a^	87.43 ± 0.66^a^
Sodium diclofenac	83.83 ± 0.21^b^	95.31 ± 0.69^b^

#### Membrane stabilizing potential (MSP)

Table 7 showed the findings of the OMW phenolic extract's membrane-stabilizing action. The extract had a higher inhibitory concentration, according to the data (IC_50_ = 87.43 ± 0.66 µg/mL) than diclofenac sodium (IC_50_ = 95.31 ± 0.69 µg/mL).

### Anticoagulant activity

The anticoagulant activity of OMW phenolic extract was evaluated in vitro by two different methods: activated partial thromboplastin time (APTT) and prothrombin time (PT)^21^.

### Endogenous coagulation pathway (APTT)

The anticoagulant activity data (Table 8) show that the extracts have a dose-dependent anticoagulant effect. The clotting time (APTT) in the presence of polyphenolic extracts of OMW and their compounds and heparin had been determined. The findings indicated that the polyphenolic extract might considerably increase the APTT (*p* ≤ 0.01). The phenolic extract of Wastewater obtained (44.77 ± 0.25 s) had a longer clotting time than the negative (28.17 ± 0.06 s) and positive (33.1 ± 0.1 s) control.

**Table 8:** Anticoagulant activity of phenolic extract of OMW.

	APTT (second)	PT (second)
Negative control	28.17 ± 0.06^c^	13.4 ± 0.1^c^
Extract	44.77 ± 0.25^a^	15.84 ± 0.12^a^
Positive control	33.1 ± 0.1^b^	14.1 ± 0.13^b^

### Exogenous coagulation pathway (PT)

The results showed that the time spent incubating polyphenolic extracts with plasma has a substantial (*p* ≤ 0.05) effect on their anticoagulant activity. From the results obtained (prothrombin time) (Table 8), it appears that the extract is capable of significantly increasing PT. The coagulation times of the phenolic extract of OMW (15.84 ± 0.12 s) was higher than that of the negative (13.4 ± 0.1 s) and positive (14.1 ± 0.13 s) controls.

### Hemolysis test

#### Anti-hemolysis activity induced by salicylic acid

**Figure 2:**
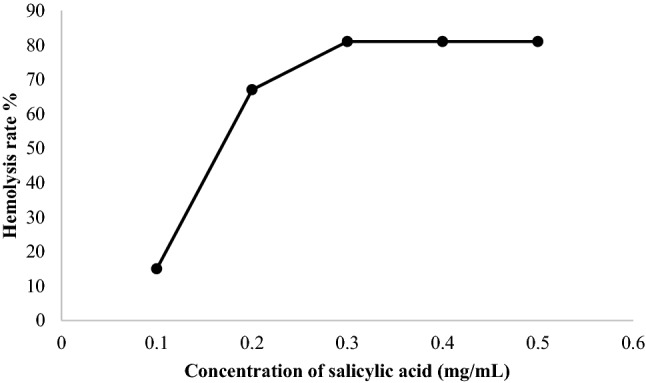
Rate of hemolysis induced by different concentrations of salicylic acid.

From the results obtained (Fig. 2), very significant hemolysis (*p* < 0.05) of (81 ± 0.71%) is obtained at a concentration of (0.3–0.5 mg/mL) of salicylic acid. Salicylic acid induced hemolysis is concentration dependent, 50% hemolysis is obtained for a concentration of ≈ 0.15 mg/mL of salicylic acid.

The statistical study also showed a highly significant erythrocyte lysis rate for the positive control and a very low significant for the negative control.

**Figure 3:**
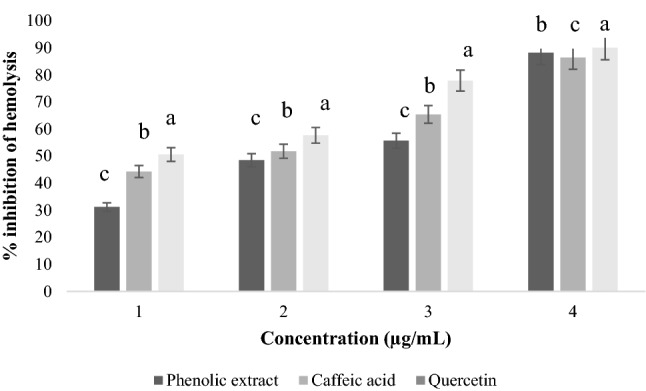
Effect of phenolic extracts of OMW on salicylic acid-induced hemolysis.

The study of the effect of phenolic extracts from OMW on hemolysis induced by salicylic acid showed that the extracts exhibit effective anti-hemolytic effects of the order of IC_50_ of (100–600 µg/mL) of extracts depending on the concentration (Fig. 3) with more or less significant differences (*p* < 0.05). The phenolic extract gave the greatest percentage inhibition of hemolysis, which is of the order of (88.2 ± 0.07%) for a concentration of 600 µg/mL. At the same maximum concentration, caffeic acid gave a percent inhibition of (90.1 ± 0.11%). Finally, an inhibition was recorded for quercetin with a percentage of (86.4 ± 0.17%).

#### Hypotonic anti-hemolysis activity

**Figure 4:**
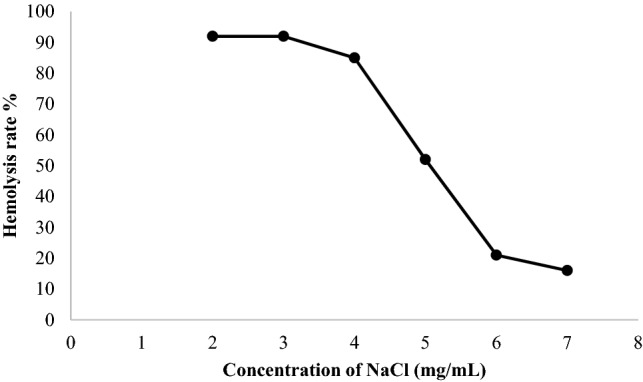
Rate of hemolysis induced by different concentrations of NaCl.

The in vitro hemolysis results carried out on human red blood cells by the hypotonic solution of NaCl (Fig. 4) showed that the maximum hemolysis was found at the concentration between 2 and 3 mg/mL with a percentage of (92 ± 0.08%).

**Figure 5:**
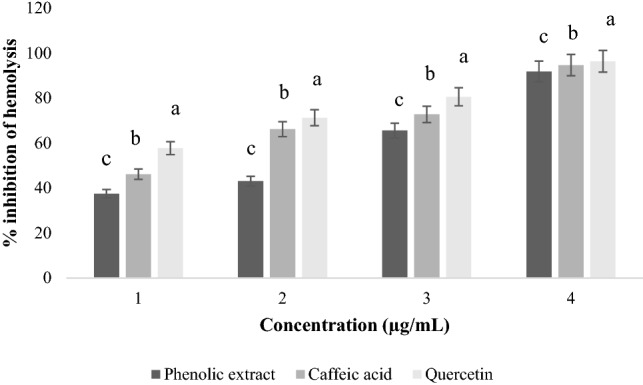
Effect of phenolic extracts of OMW on NaCl-induced hemolysis.

The inhibitory effect of phenolic extracts from OMW studied against the lysis of red blood cells showed significant differences (*p* < 0.05) between the different concentrations (Fig. 5). According to the results, the phenolic extracts of OMW showed significantly large differences depending on the concentration (*p* < 0.05). These extracts protect human red blood cells against sodium chloride-induced hemolysis. The percentage inhibition of each extract increases with the concentrations used. For example, the phenolic extract with a concentration of 800 µg/mL exhibits a maximum inhibition rate of 91.9 ± 0.17%, while at a concentration of 200 µg/mL, it exhibits a minimum rate of 37.5 ± 0.12%. The inhibition percentage the standards (quercetin and caffeic acid) present the maximum inhibition rates with a rate of 96.4 ± 0.22% for quercetin and 94.7 ± 0.08% for caffeic acid, at a concentration of 800 µg/mL.

#### Anti-hemolysis activity induced by the H_2_O_2_ radical

**Figure 6:**
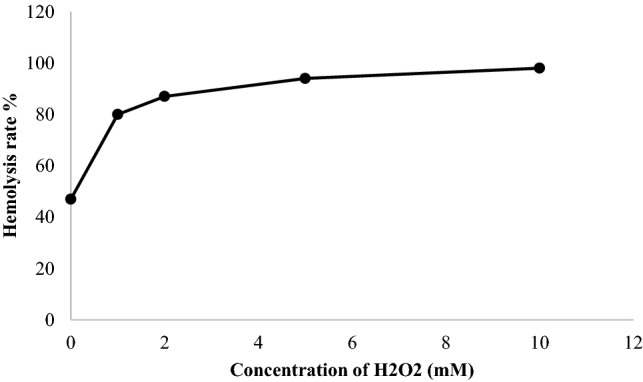
Rate of hemolysis induced by different concentrations of H_2_O_2_.

The study of the hemolytic power of hydrogen peroxide was carried out at different concentrations of H_2_O_2_, an essential step to set the concentration of H_2_O_2_, which causes optimal hemolysis. The results in Fig. 6 showed that the rate of hemolysis increases with increasing hydrogen peroxide concentration. Statistical analysis of the results of the hemolytic activity induced by the H_2_O_2_ radical showed a significant difference between the different dilutions of H_2_O_2_ and the two controls negative and positive for (*p* < 0.05) H_2_O_2_ concentrated 10 mM causes almost complete hemolysis (98 ± 2.1%). Even at a concentration of 0.1 mM of H_2_O_2_, the hemolysis rate is (74 ± 0.2%). By comparing the lysis rates at the different concentrations of H_2_O_2_ with that of the positive control, the differences are more or less significant, so the differences are highly significant with the negative control. It is observed that the osmotic fragility increases with the increase in the concentration of H_2_O_2_. A hemolysis percentage of (47.00 ± 0.06%) was obtained in the absence of H_2_O_2_ and the presence of a physiological NaCl concentration of 0.9% (w/v).

**Figure 7:**
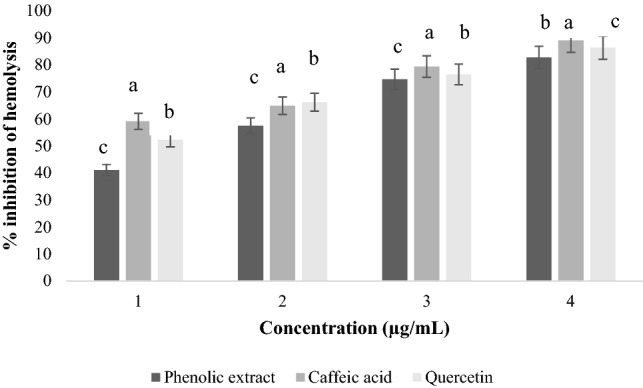
Effect of phenolic extracts of OMW on H_2_O_2_-induced hemolysis.

The results obtained on the anti-hemolytic effect of the compounds tested are presented in Fig. 7. The percent inhibition of hemolysis increases with the concentration of the compounds tested. The phenolic extract of OMW showed an inhibition of hemolysis of (41.1 ± 6.89%), (57.5 ± 4.22%), (74.7 ± 4.3%) and (82.8 ± 1.1%) at concentrations of (100, 200, 300 and 400 µg/mL), respectively. Caffeic acid shows the greatest anti-hemolytic activity with a rate of (89.1 ± 3.55%) followed by quercetin with a rate of (86.4 ± 1.24%). As for the phenolic extracts of OMW also exhibit significant anti-hemolytic activity at concentrations of (300 and 400 µg/mL) compared with the two standards caffeic acid and quercetin.

#### Effect of phenolic extract of OMW on the production of superoxide anion by neutrophils

To specify the effect of the phenolic extract of OMW on the FRO, the production of O_2_^·−^ under the same conditions with an appropriate cytochrome c probe was measured, which exclusively detects the superoxide anion. The superoxide anion production is measured; the reduction of cytochrome c is monitored at 37 °C in kinetics at 550 nm with a UVIKON 860 brand spectrophotometer.

**Figure 8:**
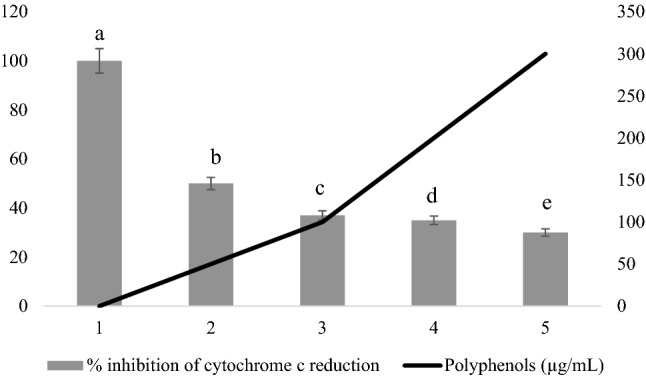
Effect of phenolic extract on the production of superoxide anion by neutrophils.

The results shown in Fig. 8 showed that the phenolic extract of OMW inhibits cytochrome c reduction in a dose-dependent manner. This result suggests that either the polyphenol extract inhibits the activity of the neutrophil NADPH oxidase, which produces this FRO precursor (superoxide anion), or it scavenges the superoxide anion. This effect can be specified by further tests using an enzyme system in which only the superoxide anion is produced, such as the xanthine and xanthine oxidase system.

## Discussion

The present study demonstrates cytotoxicity, antioxidant, anti-inflammatory, anti-hemolytic, and anticoagulant activities of phenolic compounds extracted from olive oil mill wastewater have been tested. This topic is relatively new because the aforementioned activities were studied together, as well as for the olive cultivar “Abani” from which OMW is extracted.

The composition of OMW is frequently studied in the literature, and our findings are consistent with numerous established findings. OMW is an acidic effluent with a pH range of 3–5 and a conductivity of 16.79 mS/cm. Generally, it composed of dry matter (6–17%), organic matter (4–16%), chemical oxygen demand (COD) (40–220 g/L) BOD_5_ (35–110 g/L) and the presence of several phenol-type molecules (0.5–24 g/L)^22,23^. The quality and quantity of OMW are very different and are influenced by different factors, such as type of production process, olives, use of pesticides and fertilizers, the area cultivated, weather conditions and stage of ripening olives^24^.

According to the findings, OMW is distinguished by a high concentration of phenolic components. Furthermore, the results that we obtained were higher than those obtained by^22,23^.

Several researchers were identified phenolic compounds by HPLC as^22^ that were identified ten peaks by LC–MS. They were as follows: quinic acid (23.940 ppm), Kaempferol (3.635 ppm), cirsiliol (2.352 ppm), *p*-coumaric acid (1.427 ppm), quercetin (0.470 ppm), apegenin (0.843 ppm), rutin (0.255 ppm), luteolin-7-*O*-glucoside (0.197 ppm), naringin (0.154 ppm) and quercetrin (quercetin-3-*O*-rhamnosid) (0.084 ppm).

The DPPH., ABTS.+, and FRAP free radical scavenging activity is frequently utilized to evaluate OMW phenolic compounds’ antiradical/antioxidant capability, and compared the results with several reference standards that were developed to produce more informed and perhaps required results.

Stoclet et al.^25^ showed that a strong antioxidant activity characterizes thz phenolic compounds of OMW. The in vitro antioxidant activity of bioactive compounds has received a lot of attention in the literature. The existence of oxidizing species such as free radicals or metal complexes oxidized in the presence of an extract containing antioxidants capable of preventing the formation of radicals is used in these approaches. Several studies have found that antioxidant activity is influenced by overall polyphenol content, and the type and structure of the antioxidants in the extract. Furthermore, the antioxidant capacity measured by the DPPH and ABTS assays was strongly associated with total polyphenol content.

According to^26^, at the cellular level, certain flavonoids can act on transmitting signals by protein kinases, including the expression of antioxidant and anti-inflammatory genes and vice-versa, the inhibition of oxidative and inflammatory genes.

The high antioxidant activity of the extract can be explained by the overall phenolic content and the kind of phenolic compounds present.

In addition, this strong antioxidant activity is due to the flavonoid contents, especially Kaempferol, which is the most abundant flavonoid in our extract (906.831 ± 306.164 ppm).

That means that phenolic extracts of OMW were very toxic according to the criteria of Clarkson^27^. These extracts are considered very toxic indicating the presence of powerful cytotoxic components such as heavy metals, polyphenols, etc. However, previous studies have shown that plant extract lethality against brine shrimp nauplii with a value of DC_50_ below 100 g/mL is reasonably correlated with cytotoxic and antitumor properties and may constitute potential antitumor and anticancer agents^28^.

Therefore, this "brine shrimp" larval toxicity test then constitutes a preliminary screening to determine the degree of product toxicity. Indeed, during a toxicological evaluation of plant extracts by this toxicity test, an LC50 value of < 1000 g/mL is considered bioactive^29^.

The extract showed anti-inflammatory potential due to its protein stabilizing action, but further in vivo testing is needed to validate it. Indeed, the conformation of a protein is linked to the secondary and tertiary structure; it is carried out using lower energy bonds (hydrogen bonds, electrostatic, hydrophobic and disulfide bridges), therefore fragile. Denaturation results from modifying the quaternary, tertiary and secondary structures without fragmentation of the peptide chain under the effect of various chemical or physical agents^30,31^. The denaturation of a protein causes the induction of the inflammatory reaction by producing auto-antigens, important factors for developing chronic inflammation^30^. Many flavonoids and related polyphenols have been demonstrated in recent research to have substantial antioxidant and anti-inflammatory properties. This anti-inflammatory action may be aided by the presence of these bioactive chemicals in the OMW extract. Therefore, the use of agents that can prevent protein denaturation would be helpful for the development of anti-inflammatory drugs^32^.

Stabilization of the red blood cell membrane has been used as a method to study anti-inflammatory activity in vitro because the erythrocyte membrane is analogous to the lysosomal membrane^31^. According to the findings, in comparison to diclofenac sodium, the extract demonstrated a substantial stability of the red blood cell membrane. This means that the OMW phenolic extract can effectively stabilize the lysosomal membrane. The lysosome must be stabilized to minimize the inflammatory response by limiting the release of lysosomal components such as bacterial enzymes and protease from active neutrophils. Nonsteroidal drugs such as diclofenac sodium work either by inhibiting lysosomal enzymes or by stabilizing lysosomal membranes^33^. The strong anti-inflammatory of the phenolic extract obtained in this study is probably due to Kaempferol, 4.5-di-*O*-caffeoyquinic acid, and protocatechuic acid, which are abundant in the extract.

APTT assay was used to assess the inhibition of intrinsic factors of blood coagulation pathways such as F XII, XI, V, III IX, and prekallikrein^34^. The APTT test detects VIII, IX, XI, and excitatory releasing enzymes in the endogenous coagulation pathway using brain lipids and activators rather than platelets to represent the impact of endogenous variables on coagulation time. The results obtained were in the range of those obtained from^35^. As a result, the extract examined exhibits high anticoagulant action in terms of the endogenous route.

PT assay is assessed to examine the inhibition of the extrinsic coagulation pathway, especially factors V, VII, and X^34^. To represent the influence of external variables on coagulation time, PT tests are performed by adding thromboplastin to plasma. In order to research an elongation at the level of the coagulation time, which is defined by an anticoagulant activity of the polyphenolic extracts of OMW with respect to the cascade of this pathway. Depending on the reagents employed, a typical PT lasts between 12 and 14 s. The results obtained were in the range of those obtained from^35^. As a result, the extract examined exhibits anticoagulant solid effect in the exogenous route. Heparin's anticoagulant action is due to the inhibition of endogenous coagulation enzymes when it forms a compound with anti-thrombin III.

Because thromboplastin time is a coagulation test that investigates all coagulation components in the exogenous route, it is quite possible that the anticoagulant effect of the OMW extract is related to the blockage of one of these components, which are stimulated in a cascade^36^. In addition, the phenolic extract of OMW has an anticoagulant impact on the two coagulation pathways in a dependent manner, based on the findings.

Anticoagulant medications are desperately needed since thrombotic disorders have become the leading cause of mortality. Based on the findings, the phenolic extract of OMW exhibits an anticoagulant action in vitro and might be developed as an anticoagulant medication to treat coagulation-related disorders. The extract's high anticoagulant action might be attributed to its high levels of Kaempferol, caffeic acid, and its derivatives.

Erythrocytes constitute an excellent cellular model for evaluating the effects of ROS and antioxidants^37^ due to their richness in iron and polyunsaturated fatty acids. This cell type is one of the cells most exposed to O_2_ and, therefore, to oxidation risk. Hydrogen peroxide is frequently used to trigger the formation of free radicals in red blood cells due to its ability to easily cross cell membranes^38^.

According to^39^, the exposure of human erythrocytes to oxidizing agents such as salicylic acid (aspirin precursor) only promotes hemolysis in the event of large intakes, among the side effects of Aspirin, it can cite its oxidizing effect, which can lead to the production of free radicals, the latter attacking the membrane. Aspirin is indicated in most cases in the induction of hemolytic anemia in subjects with an enzymatic deficiency in G6PD, which alters the functioning of the voice of pentose, one of the main pathways of energy metabolism which induces dysfunction sodium pump (ATPase dependent magnesium), causing a massive release of Na^+^ ions, which makes the environment hypotonic^39^. Therefore, the deficientrefore, the deficient has erythrocytes unable to resist oxidative stress because they cannot produce reduced glutathione due to lack of NADPH, therefore more protection against oxidative stress^40^.

The rate of hemolysis of red cells depended on the concentration of NaCl. In fact, the most pronounced hemolytic activity is observed in the hypotonic medium (3.5 mg/mL). In contrast, in the isotonic medium, from the concentration of 5 mg/mL, the hemolysis rate decreases until it reaches a minimum at the concentration of 7 mg/mL. If an erythrocyte is placed in a hypotonic medium, water enters the cell and gradually creates a deformation of the blood cells; beyond a certain threshold, the membrane bursts and intracellular hemoglobin passes into the medium exterior (hemolysis)^41^.

The results showed that the osmotic fragility increases with increased oxidative stress induced by hydrogen peroxide. Low-concentration hydrogen peroxide-induced hemolysis results from destructive oxidative damage to the cytoplasmic membrane following lipid peroxidation of Polyunsaturated Fatty Acids (PUFA) present therein^42^. When H_2_O_2_ crosses this membrane, it can cause the breakdown of hemoglobin hem and Fe^2+^ ions, which generates the hydroxyl radical, which is very reactive by the Fenton reaction. These two radicals induce a chain of lipid peroxidation leading to the lysis of erythrocytes^43^. On the other hand, at high concentrations, the rate of hemolysis decreases, which may be linked to the insolubility of hemoglobin following its polymerization and the aggregation of erythrocytes^43^.

The results of anti-hemolytic tests have shown that the phenolic extract exerts a protective effect on red blood cells and provides protection against hemolysis of red blood cells and denaturation of hemoglobin with a dose dependent.

From work carried out, it is possible to suggest that the anti-hemolytic effect of the phenolic extracts of OMW is due to the prevention of methemoglobin formation, following the trapping of hydrogen peroxide. This phenomenon prevents or decreases the formation of the hydroxyl radical and therefore prevents oxidative damage to membrane constituents, thus preventing the induction of hemolysis. Another possible protection mechanism would be the trapping of the hydroxyl radicals formed and a chelating action of the metals of these molecules.

## Methods

### Ethics statement

All experiments were conducted in accordance with the guidelines of the Declaration of the World Medical Association of Helsinki. The experiments were approving by the scientific committee of the Faculty of Nature and Life Sciences, Abbes Laghrour University of Khenchela, Algeria. They were performed in accordance with relevant guidelines and regulations. The blood donation for this in vitro study was approved by the Ethics Committee of the Central Laboratory of Ahmed Ben Bella Hospital Khenchela, Algeria. Written consent to participate and approval for publication was obtained from each volunteer. Informed consent was obtained from all subjects.

### Physicochemical properties

The olive oil mill wastewater (OMW) from Abani variety was obtained from a modern olive mill situated in Khenchela, eastern Algeria, in November 2019. It was collected directly from the decanter, frozen immediately and kept at − 20 °C until use. Standard Methods^44^ were used to measure pH, electrical conductivity (EC), dry matter (DM), total suspended solids (TSS), organic matter (OM), mineral matter (MM), biological oxygen demand (BOD_5_), and chemical oxygen demand (COD). The pH level was measured using a pH meter (AdwaAD1000). Electrical Conductivity (EC) was determined by conductivity meter type (inoLab WTW). Dry matter content (DM) was measured by drying at 105 °C for 24 h. Organic matter (OM) was calculated by the difference between the dry weight of the OMW and its weight after the calcination. Mineral matter (MM) was determined by weighing after ignition in a muffle furnace type (Nabertherm) at 550 °C, for 24 h. The chemical oxygen demand (COD) was determined using potassium dichromate, as described by BOD_5_ (biological oxygen demand) is determined by the respirometric method. Analyzes were carried out in triplicate.

### Polyphenol extraction methods

The phenolic compounds were extracted using the maceration method. 1 g of OMW powder was mixed with 10 mL of pure methanol. Then, vortexed for 15 min and kept to macerate overnight at 4 °C in the dark. After maceration, filtering using filter paper is performed. The macerate was collected and added to 10 mL of methanol (90%) for a second time; the combination was vortexed for 15 min before being left to macerate for 1 h. The two filtrates are mixed and filtered through sodium sulfate-containing cellulose paper. The solution was condensed in a rotary evaporator (HAHNVAPOR) at 40 °C, and the dry material was stored.

### Total phenolic content (TPC)

The total phenolic content was determined following the Folin–Ciocalteu method^45^. Taking 125 µL of the extract diluted 100 times is put in the presence of 500 µL of distilled water and 125 µL of the FCR After stirring and standing for 3 min, 1250 µL of a 7% CO_3_Na_2_ solution was added to the mixture. The volume of the mixture was adjusted to 3 mL with ultrapure water and then left in the dark at room temperature for 90 min. The results were expressed as milligrams of gallic acid equivalents per milliliter of extract (mg GAE/mL). Gallic acid calibration curve was used to quantify the total phenolic content (TPC) of extracts (y = 0.0046 x + 0.0108, R^2^ = 0.9967).

### Total flavonoids content (TFC)

The quantification of total flavonoids content was performed by the method of^46^. To a dose of 250 µL of the extract diluted 100 times with methanol, is added 75 µL of a 5% NaNO_2_ solution. After 6 min of incubation at room temperature, 150 µL of an aluminum chloride solution AlCl_3_ was added to the mixture. After 5 min of incubation at room temperature, 500 µL of sodium hydroxide was added to the mixture and then the volume was adjusted to 2500 µL with distilled water. The results were expressed as milligrams rutin equivalents per milliliter of extract (mg RE/mL). The total flavonoids content (TFC) was calculated following the calibration curve prepared from rutin (y = 0.0103 x + 0.0061, R^2^ = 0.9963).

### Tannin condensed content (TCC)

The quantification of condensed content was performed according to the method of^47^ by reaction with vanillin in the presence of sulfuric acid. A volume of 0.5 mL intake of the suitably diluted extract is mixed with 2 mL of 1% vanillin and then added with 2 mL of concentrated sulfuric acid. After homogenization, the mixture is incubated at room temperature. The results were expressed as milligrams of tannic acid equivalent per milliliter of extract (mg TAE/mL). The tannins condensed content (TCC) was calculated using tannic acid calibration curve (y = 0.0066 x + 0.0113, R^2^ = 0.9969).

### LC–MS separation and identification of phenolic compounds

The OMW extracts were analysed using a Shimadzu UFLC XR system consisted of an electrospray ionization source (ESI) equipped with two LC-20ADXR solvent delivery units, a SIL-20AXR autosampler, an SCL-10A system controller, a CTO-20 AC column oven, a DGU-20AS degasser (Shimadzu, Kyoto, Japan). A volume of 5 μL each extract was injected at 0.5 mL/min to a Discover BIO Wide Pore C18 column (150 mm × 3 mm, 3 μm) at 40 °C and separated with two mobile phases: A (0.1% formic acid in water v/v) and B (0.1% formic acid in methanol v/v) following the programmed linear gradient elution : 0–14 min, from 10 to 20% B; 14–27 min, from 20 to 55% B; 27–37 min, from 55 to 100% B; 37–45 min, 100% B; and 45–50 min 10% B^48,49^. The ionization mode was negative and The ESI conditions were set as follows: capillary voltage of − 3.5 v, a nebulizing gas flow of 1.5 L/min, a dry gas flow rate of 15 L/min, a DL (dissolving line) temperature of 280 °C, a block source temperature of 400 °C, and a voltage detector of 1.35 V. Compounds were identified by comparing their retention time and mass spectra with those of reference standards. The validation of the method was determined as detailed in^50^.

### Antioxidant assays

#### DPPH free radical-scavenging activity

The antioxidant activity of different extractions was evaluated following^51^ method using the free radical DPPH (2,2-diphenyl-1-picrylhydrazyl) with some adjustment. A test sample of 0.5 mL of the extract at different concentrations is mixed with 0.5 mL of a solution of DPPH (0.2 mM in methanol). After vigorous shaking of the mixture, it is left to stand for 30 min in the dark. The results were given as 50% inhibition concentration (IC_50_) and compared with the antioxidant standards (BHT, Ascorbic acid and rutin).

#### ABTS^+^ free radical scavenging activity

The ABTS (2,2-Azino-bis-3-ethyl benzothiazoline-6-sulfonic acid) scavenging activity was determined according to the method of^51^ with some adjustment. A volume of 10 μL of the extract is added to a volume of 990 μL of ABTS solution. The discoloration relative to the control, containing ABTS^+^ and the solvent (ethanol), is measured with a spectrophotometer at 734 nm after 30 min of incubation in the dark. The results were given as 50% inhibition concentration (IC_50_) and compared with the antioxidant standards (BHT, Ascorbic acid, and rutin).

#### FRAP ferric reducing antioxidant power

The FRAP activity was evaluated following^51^ with some adjustment. It consists of mixing 1 mL of each solution of extracts or of the standard antioxidant at different concentrations with 1 mL of phosphate buffer (0.2 M, pH 6, 6) and 1 mL of a 1% solution of potassium ferricyanide[K_3_Fe (CN)_6_]). The mixture obtained is incubated at 50 °C for 20 min, and then 1 mL of 10% trichloroacetic acid (CCl_3_COOH) is added to stop the reaction. The mixture is centrifuged at 2000*g* for 10 min. To 1 mL of the supernatant are added 1 mL of distilled water and 0.5 mL of 0.1% iron chloride (FeCl_3_). The reaction medium is incubated at room temperature for 10 min. The results were given as 50% inhibition concentration (IC_50_) and compared with the antioxidant standards (BHT, Ascorbic acid, and rutin).

#### Brine shrimp cytotoxicity test

The brine shrimp lethality bioassay was performed using the method of^29^. *Artemia nauplii* were obtained by hatching brine shrimp eggs (*Artemia salina*) in artificial seawater (3.8% NaCl solution) for 48 h. The dissolution of 30 mg of OMW was carried out in 3 mL of artificial seawater containing 20% ​​DMSO to give a concentration of 10 μg/mL. From this solution 0.1, 5, 10, 20, 40, and 80 μL were transferred to each 10 mL vial and using artificial seawater the volume was adjusted to 10 mL per artificial seawater. *Artemia nauplii* were cultivated in these solutions and their mortality was observed after 24 h. The number of surviving larvae is counted in each tube and the mortality is calculated at each concentration as follows:

$$\% \;{\text{Deaths}}{\mkern 1mu} = {\mkern 1mu} (\% \;{\text{test deaths}}{\mkern 1mu} - {\mkern 1mu} \% \;{\text{control deaths}})/(100{\mkern 1mu} - {\mkern 1mu} \% \;{\text{control deaths}}).$$

The control mortality should not exceed 15%. The artificial seawater medium containing DMSO used for the analysis was used as a negative control. K_2_Cr_2_O_7_ was used as a standard in this test.

The 50% Death Concentration (DC50) reflecting the toxicity of the products is estimated as: (−)Toxicity when DC50 ≥ 100 μg/mL, (+) Toxicity when 100 μg/mL > DC50 ≥ 50 μg/mL, (++) Toxicity when 50 μg/mL > DC50 ≥ 10 μg/mL, and (+++) Toxicity when DC50 < 10 μg/mL.

The number of dead and alive nauplii was recorded after 24 h. Nauplii were considered dead if no internal or external movement was observed within 30 s. The percent mortality of salted shrimp and the LC 50 (median lethal concentration) was then calculated. This was done by plotting the percent mortality against the logarithm of the extract concentration. The LC 50 value was derived from the regression equation^29^.

### Cytotoxicity test of the phenolic extract of OMW on human neutrophils

#### Isolation of neutrophils by the Dextran–Ficoll method

The purpose of this method is to separate blood cells from fresh blood taken on an anticoagulant. The principle is based on the separation of blood components according to their density. The number of neutrophils recovered from a 400 mL blood bag after isolation ranges from 6 to 10 × 10^8^ neutrophils. Isolation of Polynuclear Neutrophils begins with mixing one volume of blood with one volume of 2% Dextran-T500 (prepared in advance in 0.9% NaCl and filtered). After 30 min incubation at room temperature, the majority of erythrocytes precipitate at the bottom of the tube. The upper phase contains the white blood cells. The latter is collected in clean tubes and the red blood cells of the lower phase are discarded. The phase containing the white blood cells is centrifuged for 8 min at 400 g at 22 °C. The pellet is suspended a second time in phosphate buffer, then deposited on a Ficoll cushion and centrifuged for 30 min at 400*g* and at 22 °C. After this step, three phases are distinguished, the pellet corresponds to the granulocytes (eosinophils, neutrophils and basophils) with the contaminating erythrocytes, the mononuclear cells (monocytes and lymphocytes) form a ring between the pellet and the supernatant is a mixture of plasma, buffer phosphate and Ficoll. Lysis of residual erythrocytes is carried out by adding a cold 0.2% NaCl hypotonic solution to the pellet, mixing for 40 s, isotonicity is restored by adding the same volume of 1.6% NaCl. The solution is buffered by adding cold phosphate buffer. The neutrophils are recovered in a small volume of phosphate buffer, after centrifugation for 8 min at 400*g* and 4 °C. Neutrophils are stored at 4 °C until use.

#### Neutrophil viability and purity test

The neutrophil viability test is performed by the Trypan blue exclusion test, which stains dead cells blue. While the purity of Polynuclear Neutrophils is checked with another dye, crystal violet stains the nuclei of cells, making it possible to distinguish the polylobed nucleus characteristic of neutrophils from other cell nuclei. The count is done on a Malassez slide by light microscopy at magnification × 40. Their effect has been verified on the viability of neutrophils, this test is very important if one plans to use these molecules in humans. To test the purified extracts, the purity of the freshly isolated neutrophils is checked with crystal violet (dye-based on gentian violet and acetic acid) and they are counted. The cells are resuspended in phosphate buffer and stored at 4 °C before using them. Before testing the extracts of the polyphenols on the neutrophils, it was been checked the cytotoxicity of the product, by incubating the neutrophils in the presence of high concentrations of polyphenols ranging from 0 to 300 µg/mL for 30 min, the dead cells allow the blue of Trypan, which stains them blue, unlike living cells which remain transparent.

### Anti-inflammatory activity

#### Inhibition of protein denaturation (IPD)

It is determined by the method described by^52^ with slight modifications. The concept is that the phenolic extract of OMW inhibits denaturation of BSA induced by heat (72 °C) 0.1 mL of each concentration of extract added to 1 mL of 0.2% BSA solution prepared in Tris–HCl pH 6.6, then incubated at 37 °C for 15 min then in a water bath at 72 °C for 5 min. After cooling, the turbidity is measured at 600 nm in a cell spectrophotometer (SPECORD 210 plus). Diclofenac sodium standard (injectable form) was produced using the same technique in ultra-pure distilled water from a 500-ppm mother solution, with distilled water serving as a negative control.

#### Membrane stabilizing potential (MSP)

It was measured according to^53^. An equivalent volume of blood was collected from healthy human volunteers who had not taken any NSAIDs for two weeks before blood collection and combined with an equal volume of sterile Alsever solution. This blood solution was centrifuged for 10 min at 3000 rpm, the packed cells were separated and washed with iso-saline solution, and a 10% (v/v) suspension was made using an iso-saline solution.

1 mL phosphate-buffered saline, 0.5 mL 10% blood suspension, 0.5 mL phenolic extract of OMW with various concentrations, and 2 mL hypotonic saline make up the dose combination. All test mixtures were incubated at 37 °C for 30 min and then centrifuged at 3000 rpm for 20 min. The hemoglobin concentration was measured using a spectrophotometric measurement at 560 nm after the supernatant was separated.

The negative control was distilled water and the positive control was diclofenac sodium at the final concentration. The IC_50_ was measured once again using a graph that showed inhibition at various doses.

### Anticoagulant activity in vitro

#### Endogenous coagulation pathway (APTT)

Activating partial thromboplastin time (APTT) was determined according to^29^. A platelet plasma pool comprises a plasma combination from ten healthy, untreated individuals with normal APTT and PT.

The activity of the phenolic extract was established on a volume of 100 μL whose plasma is 90 μL was mixed with 10 μL of extract. After 15 min of incubation at 37 °C 100 μL cephalin kaolin was added to the mixture, which was re-incubated for 3 min with agitation at 37 °C. Using a coagulometer, the coagulation time was measured by adding 100 µL of warmed calcium chloride (0.025 M). In parallel, a positive control of calciparine (unfractionated heparin) and a negative control test (substituting the samples with a 0.9% NaCl solution) were performed under identical circumstances. An increase in APTT in the presence of polyphenols compared to the negative control implies an anticoagulant impact at this route level. Clotting time was determined by an automatic coagulation analysis system (Coa DATA 4004).

#### Exogenous coagulation pathway (PT)

Prothrombin time (PT) was determined according to the protocol described by^54^. The coagulation time of citrated plasma in the presence of an excess of calcium thromboplastin is measured in this activity using platelet-poor plasma in the presence of calcium thromboplastin. The phenol extract (90 and 10 µL, respectively) was combined with 100 µL of platelet-poor plasma that had been warmed for 2 min at 37 °C. After 15 min of incubation at 37 °C, 200 µL of calcium thromboplastin was added to the mixture, which had been warmed for at least 15 min at 37 °C. Coagulation time was determined by an automatic coagulation analysis system (CoaDATA 4004).

### Study of antioxidant activity on a cell model

#### Hemolysis test

The anti-hemolytic effect of plant extracts is evaluated in vitro using the Erythrocyte model. The latter is easily isolated from blood and its membrane shows similarities to other cell membranes^55^.

#### Preparation of the erythrocyte suspension

The blood used to prepare the erythrocyte suspensions was taken from healthy people in heparin tubes. Serological analyses were carried out to exclude any risk of contamination of any pathology. After centrifugation of the blood at 3000 rpm/5 min, the recovered pellet is washed 3 times with the Phosphate Buffered Saline (PBS) solution formed from 10 mM potassium phosphate buffer, pH = 7.4 and 154 mM of NaCl. Each wash consists of a suspension of the cells in iso-saline PBS and centrifugation at 3000 rpm/5 min. After the last centrifugation, the pellet is suspended for the second time again in a solution of iso-saline PBS at the rate of 1 volume of the pellet and 9 volumes of PBS, thus obtaining a hematocrit at 10%^56^.

#### Development of in vitro induced hemolysis tests

The exposure of red blood cells (RBC) to certain physicochemical parameters such as the hypotonic medium, the use of a membrane disruptor such as detergents or reactive oxygen species, causes a rupture of its cytoplasmic membrane thus causing the release of the hemoglobin, which will then be determined by visible absorbance spectrophotometry at 540 nm. To test the anti-hemolytic effect of the phenolic extracts of OMW, tests on an erythrocyte model with hemolysis induced by three different agents (hypotonic medium, salicylic acid and H_2_O_2_) were carried out.

#### Induction with salicylic acid

In test tubes each containing 4.5 mL of hypotonic NaCl (4.5 mg/mL), 50 μL of salicylic acid in different concentrations (0.1, 0.2, 0.3, 0.4 and 0.5 mg/mL) were added. The control tube receives the same volume of PBS buffer, and then a volume of 500 μL of the erythrocyte suspension is added to each tube. After that, the tubes are homogenized, incubated at 37 °C for 30 min in a water bath, and centrifuged at 3000 rpm for 5 min. The absorbance is then measured at 540 nm^39^.

#### Hypotonic induction

To determine the concentration of NaCl, which causes the lysis of red blood cells, 100 μL of the erythrocyte suspension (10%) were added to 5 mL of NaCl at different concentrations (2, 3, 4, 5, 6 and 7 mg/mL), as well as a negative (Isotonic NaCl 9 mg/mL) and positive (distilled water) control. After incubation for 30 min at room temperature, the mixture was centrifuged at 3000 rpm/10 min and the DO was read at 540 nm^57^.

#### Induction by hydrogen peroxide H_2_O_2_

A volume of 500 μL of H_2_O_2_ at different dilutions (0, 1, 2, 5 and 10 mM) were mixed with a volume of 250 μL of the suspension of red blood cells. After 3 h of incubation at 37 °C, PBS was added and then the mixture was subjected to centrifugation for 10 min with a speed of 3000 rpm. The absorbance of the supernatant was read at 540 nm. Controls were prepared by replacing H_2_O_2_ with distilled water for the positive control and with PBS for the negative control^48^.

The percent hemolysis for all tests was calculated using the following formula^55^:

$$\% {\text{ hemolysis}}\, = \,\left( {{\text{Absorbance of the test}}/{\text{Absorbance of the control}}} \right)\, \times \,{1}00.$$

#### Measurement of the oxidative explosion of polynuclear neutrophils by the cytochrome c reduction technique

The production of O_2_^·−^ by activated neutrophils is measured by the cytochrome c reduction technique^49^. The principle of this test is based on the use of oxidized cytochrome c "Fe^3+^" in the presence of a source producing superoxide anions (O_2_^·−^) such as neutrophils or by an acellular system composed of xanthine/hypoxanthine and xanthine oxidase. In the presence of this very reactive and unstable O_2_^·−^ radical, cytochrome c is reduced to Fe^2+^. This test exclusively detects the extracellular superoxide anion because cytochrome c does not cross the cell membrane; the reduced cytochrome is measured by spectrophotometry at 550 nm. In practice, neutrophils at 10^6^ cells/mL in phosphate buffer are pretreated with increasing concentrations of polyphenols (0, 50, 100, 200 and 300) µg/mL for 10 min at 37 °C. The cells are incubated in the presence of cytochrome c at 1 mg/mL final and then stimulated with Phorbol Myristate Acetate (PMA) (100 ng/mL) and the absorbance is measured at 550 nm using a thermostated spectrophotometer brand UVIKON 860. The production of O_2_^·−^ is measured for 10 min and the results are expressed in nanomoles of O_2_^·−^ produced per minute and per million neutrophils using Beer Lambert's law: A = ε. C. l, where ε is the molar extinction coefficient (l. Mol^−1^. Cm^−1^), A being the absorbance (without unit), C is the concentration of the solute (mol/L).

### Statistical study

Data obtained were presented as mean ± SD of three dependent determinations. Significant differences between means of total phenolic, total flavonoids, tannins and LC–MS analysis results were determined by Student t-test, and *p* values (< 0.05) were regarded as significant. Results of antioxidant, anti-inflammatory and anticoagulant activities were subjected to statistical analysis of variance (ANOVA) using ECXEL STAT (version 2014) package at *p* < 0.05 significant levels.

## Conclusions

The current study aims to determine cytotoxicity, antioxidant, anti-inflammatory, anti-hemolysis, and anticoagulant activities, of phenolic compounds extracted from OMW from Abani cultivar. Because of few scientific works have been carried out on the enhancement and evaluation of the biological potential of polyphenols of OMW, this topic is considered that be relatively new. It shows for the first time that phenolic extracts of OMW have anti-hemolytic and anti-inflammatory properties. In addition, it demonstrates that they possess potent dose-dependent toxicity was observed in brine shrimp lethality assay, antioxidant and anticoagulant activity, and can stabilize human erythrocyte membranes in a dose-dependent manner. Chemical examination reveals the presence of polyphenols, flavonoids and tannins that may be responsible for the aforementioned properties. The major phenolic compound revealed by LC–MS is Kaempferol. The overall results of the current study demonstrate that OMW offers very promising prospects for valorizing polyphenols and reducing their polluting impact on the environment and OMW bioactivities increase the valorization of these by-products. Concluding, the obtained results are interesting, however, the mechanisms underlying the observed effects are unknown. It would therefore be wise in the future to deepen the phytochemical study of this effluent by trying to identify and purify the phenolic compounds responsible for these biological activities and to test them in vivo. In addition, more extensive research is needed to identify the secondary metabolites responsible for the reported biological activities and discover the underlying mechanism behind these therapeutic potentials.

## Abbreviations

OMW: Olive oil mill wastewater
LC–MS: Liquid chromatography–mass spectrometry
DPPH: 2,2-Diphenyl-1-picrylhydrazyl
ABTS^+^: 2,2′-Azino-bis (3-ethylbenzothiazoline-6-sulfonic acid)
FRAP: Ferric reducing ability of plasma
IC_50_: Half-maximal inhibitory concentration
IDP: Inhibition of protein denaturation
MSP: Membrane-stabilizing potential
APTT: Endogenous coagulation pathway
PT: Exogenous coagulation pathway
EC: Electrical conductivity
DM: Dry matter
OM: Organic matter
MM: Mineral matter
BOD_5_: Biological oxygen demand
COD: Chemical oxygen demand
TPC: Total phenolic content
TFC: Total flavonoids content
TCC: Tannin condensed content
DC_50_: Death concentration
LC_50_: Median lethal concentration
PBS: Phosphate buffered saline
RBC: Red blood cells
PMA: Phorbol myristate acetate
PUFA: Polyunsaturated fatty acids
DMSO: Dimethyl sulfoxide
ROS: Reactive oxygen species
FRO: Free radicals of oxygen
NADPH: Nicotinamide adenine dinucleotide phosphate
